# Children view own-age faces qualitatively differently to other-age faces

**DOI:** 10.1080/20445911.2016.1164710

**Published:** 2016-03-29

**Authors:** Peter J. Hills, Susan F. L. Willis

**Affiliations:** ^a^ Department of Psychology, Bournemouth University, Dorset, UK; ^b^ Department of Psychology, Anglia Ruskin University, Cambridge, UK

**Keywords:** Eye-tracking, perceptual expertise, own-age bias, face recognition, pupillometry

## Abstract

Like most own-group biases in face recognition, the own-age bias (OAB) is thought to be based either on perceptual expertise or socio-cognitive motivational mechanisms [Wolff, N., Kemter, K., Schweinberger, S. R., & Wiese, H. (2013). What drives social in-group biases in face recognition memory? ERP evidence from the own-gender bias. *Social Cognitive and Affective Neuroscience*. doi:10.1093/scan/nst024]. The present study employed a recognition paradigm with eye-tracking in order to assess whether participants actively viewed faces of their own-age differently to that of other-age faces. The results indicated a significant OAB (superior recognition for own-age relative to other-age faces), provided that they were upright, indicative of expertise being employed for the recognition of own-age faces. However, the eye-tracking results indicate that viewing other-age faces was qualitatively different to the viewing of own-age faces, with more nose fixations for other-age faces. These results are interpreted as supporting the socio-cognitive model of the OAB.

The own-age bias (OAB) in face recognition is typically demonstrated by superior recognition for own-age faces relative to other-age faces (e.g. Anastasi & Rhodes, 2006; Harrison & Hole, 2009; Hills & Lewis, 2011; Kuefner, Macchi Cassia, Picozzi, & Bricolo, 2008; Rhodes & Anastasi, 2012). While this effect appears sensitive to task instructions (He, Ebner, & Johnson, 2011), it has been found in young adults (Wiese, Schweinberger, & Hansen, 2008), older adults (Firestone, Turk-Browne, & Ryan, 2007), and children (Anastasi & Rhodes, 2005). Similar to the own-ethnicity bias, there are two main theories explaining the OAB: that of perceptual expertise and that of socio-cognitive motivation (Wolff, Kemter, Schweinberger, & Wiese, 2013; Wiese, Komes, & Schweinberger, 2013).

In short, the perceptual expertise account of the OAB suggests that due to extensive experience with faces of our own age, participants utilise expert face processing skills for own-age faces and inexpert processing for other-age faces (e.g. Macchi Cassia, Picozzi, Kuefner, & Casati, 2009). As evidence for this, Kuefner et al. (2008) demonstrated that young adults did not show a face-inversion effect (an effect often used as evidence for expert processing, Valentine, 1988) for other-age faces but did for own-age faces (see also Hills & Lewis, 2011). Participants with current experience with other-age faces do show the face-inversion effect for those other-age faces (Harrison & Hole, 2009; Kuefner et al., 2008; Macchi Cassia et al., 2009). The socio-cognitive motivational account of the OAB is based on Sporer’s (2001) in-group/out-group model of the own-ethnicity bias. In this account, faces are quickly classified according to their group status, either as own-group or other-group. Own-group faces are subsequently attended to specifically and processed in a deep and effortful manner, whereas other-group faces are processed in a shallow manner.

Eye-movement measures can provide evidence supporting one of the accounts. Eye-movements provide an index of the allocation of visual attention (Findlay & Gilchrist, 2003; Yarbus, 1965) and are functional in face learning (Althoff & Cohen, 1999). Enhanced attention to faces may be reflected in the way that faces are viewed (Buswell, 1935; Isaacowitz, Wadlinger, Goren, & Wilson, 2006). Indeed, it has been shown that there are differences in the way that own- and other-ethnicity faces are viewed, with more fixations to the nose in other-ethnicity faces than own-ethnicity faces (Goldinger, Papesh, & He, 2009, but see e.g. Caldara, Zhou, & Miellet, 2010) suggesting that the way the faces are categorised affects the way they are viewed. It has also been established that young and older adults look at own-age faces more than other-age faces (Ebner, He, & Johnson, 2011; He et al., 2011), however, during expression recognition tasks they do not look at different features. Firestone et al. (2007) have also indicated potential OABs in scanning behaviour when viewing faces, with a trend for more transitions between areas of interest (AOIs) (eyes, nose, and mouth) in own-age faces than other-age faces. These results indicate that there may be eye-movement differences when viewing own- and other-age faces indicating differential processing employed. However, the precise differences when viewing own- and other-age faces have not been established and this effect has not been tested in children.

Goldinger et al. (2009) proposed that pupillometry could be used as a metric for measuring the effort put in during the encoding of faces. When people engage in more cognitive effort, their pupils dilate (Porter, Troscianko, & Gilchrist, 2007). Goldinger et al. (2009) found that when viewing other-ethnicity faces, participants’ pupils were more dilated than when processing own-ethnicity faces, suggesting that they were engaging in more effortful processing for other-ethnicity faces than own-ethnicity faces. While acknowledging the limitations with pupillometry (the fact that the biggest impact on pupil width is the luminance of the image, Porter et al., 2007), we believe this will indicate how much effort children put into recognising own- and other-age faces.

Taken together, the research summarised thus far indicates that the OAB in face recognition may be based on perceptual or socio-cognitive mechanisms. Eye-tracking differences should reveal whether participants are engaging in differential processing for own- versus other-age faces. This has yet to be established in a face recognition paradigm and has never been tested in school-aged children. We, therefore, ran a face recognition study using eye-tracking on school-aged children. We tested their recognition of own- and other-aged upright and inverted faces, while recording the eye movements to explore expertise and motivation in the OAB. We hypothesise that if participants code own- and other-age faces differently, there will be eye-tracking and pupillometry differences revealed through an interaction between face age and feature viewed. However, if they do not change the processing styles, rather the OAB is based on perceptual expertise, our participants will show a larger face-inversion effect for own-age relative to other-age faces.

## Method

### Participants

Participants were: 43 (19 female) 6- to 11-year-old ethnically White children (mean age: 8 years 7 months) recruited from a sample who returned consent forms to their school. All of the children had normal or corrected-to-normal vision and were considered typically developing by their schools.

### Materials

Two photographs of 44 children were collected by one of the authors (SFLW). These were all taken with the same camera in front of the same white background, with light from above and to the front. The two photographs were taken shortly after each after, but the children were making a slightly different expression in the second photograph. These photographs were taken of children from the same school-year groups but a different primary school in Cambridgeshire, therefore they were matched for age to the participants. All participants reported that the faces were unfamiliar to themselves. All faces displayed a happy-neutral expression and no extraneous features (such as clothing). Images were equated for luminance and mean root contrast in Photoshop and any background removed. An inverted version of each face was created by rotating the faces 180°. Images were constrained to the proportions 428 × 368 pixels in size, subtending to an approximate visual angle of 10.19° × 9.86°, with the resolution 72 dpi. Five AOIs (similar to Goldinger et al., 2009) were mapped out on the stimuli individually. These were: forehead and hair, eyes, nose, mouth, and chin and cheeks. These areas were not visible to participants.

The faces were displayed on a white background, in the centre of a 15″ (1280 × 800 pixels) LCD colour monitor, using ClearView v2.7.0. Eye movements were recorded using a Tobii X50 eye-tracker (Falls Church, VA), with embedded infrared cameras with a sampling rate of 50 Hz. The eye-tracker was positioned in front of the laptop, under the screen, 60 cm from the participant. A fixation was defined as the eyes remaining in the same 30 pixel area for 100 ms (see Goldinger et al., 2009). If the eyes left the region, but returned within 100 ms, it was considered to be the same gaze. These settings were based on the defaults for the Tobii eye-tracker.

### Design

A 2 × 2 within-subjects design was employed with the factors: face group (own- and other-age) and orientation (upright and inverted). Age group was defined as faces that were in the same school year as the participant were considered own-age and faces that were not in the same school year were considered as other-age (see Hills & Lewis, 2011). This, by necessity, creates an unequal number of stimuli in each condition. This is controlled for by using the appropriate sums of squares in all calculations of recognition accuracy. Faces were counterbalanced such that each appeared as a target and a distractor an approximately equal number of times and inverted and upright an equal number of times. The dependent variables were recognition accuracy, measured using the Signal Detection Theory (e.g. Swets, 1966) measure, *d′*. Eye-tracking measures were also taken. These were total duration of fixation to each AOI, number of fixations to each AOI, and pupil width.

In order to analyse the eye-tracking data, we employed a procedure similar to Bindemann, Scheepers, and Burton (2009): area-normalised scores were calculated by dividing the proportion of fixations (or durations) by the proportion of the screen the AOI occupied. In effect, this transformation equates the size of each AOI so that a score of one indicates the AOI is scanned at random whereas a score significantly higher than one indicates that the region is specifically scanned (Fletcher-Watson, Findlay, Leekam, & Benson, 2008). It also equates the duration that each stimulus was viewed for. An analysis on the raw data revealed a similar pattern of results for the internal features but larger proportion of fixations to external features (though no differences across conditions).

### Procedure

We employed an established recognition procedure (see e.g. Anastasi & Rhodes, 2005; Harrison & Hole, 2009; Hills & Lewis, 2011; Wiese et al., 2008). Participants were tested individually in a room in their school. A teacher was present during all testing. The experimenter controlled the computer screen, but was unaware of what the screen was displayed since it was swivelled away from her. This procedure was used to ensure that the participants fully engaged with the task by giving verbal responses and remaining as still as possible in order to record accurate eye-movement data. Participants sat approximately 70 cm away from the computer screen. The subsequent experiment involved four consecutive phases: calibration, learning, distraction, recalibration, and test.

Participants’ eyes were first calibrated to the eye-tracker. Calibration was achieved by asking the child to follow a blue dot that moved to five pseudo-random points on the screen. Calibration was successful for all participants at the first or second attempt.

In the learning phase, participants were shown half (22) of the faces, of which half were own age and half were other age (five of each type of face were inverted). These were presented sequentially in a random order. Participants were asked to verbally state whether they thought the face was “weird looking or not” in order to ensure they attended to each face (they were not told to remember the faces). The presentation lasted 2 s. There was a blank inter-trial-interval of 150 ms. There were no significant differences across participant groups for making these judgements. Participants were not informed of the subsequent recognition test, therefore the learning was incidental.

The distraction phase involved a series of unrelated filler questions, typically lasting 30 s. Participants were then recalibrated to the eye-tracker. This involved the same calibration procedure.

Following this, the participants were given the test phase. In this, the participants saw all of the faces they had seen before and 22 new faces (half own-age and half other-age; six of each upright) and had to make a verbal old/new recognition judgement to each face. The faces were presented sequentially in a random order. Each presentation was response terminated. Between each face a grey mask was presented for 150 ms.

## Results

The recognition accuracy measure, *d′,* was calculated using the Macmillan and Creelman (2005) method. For the present study, *d′* values typically ranged from 0 to 4, whereby 0 is recognition at chance levels and 4 is near-perfect recognition. The recognition accuracy data shown in Table 1 were subjected to a 2 × 2 within-subjects ANOVA with the factors: face age (own- and other-age) and orientation (upright and inverted). This revealed a main effect of face age, *F*(1, 42) = 4.63, MSE = 0.74, *p* = .037, 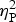 = .10, in which own-age faces (*M* = 1.25, SE = 0.06) were recognised more accurately than other-age faces (*M* = 0.97, SE = 0.13). There was also a main effect of orientation, *F*(1, 42) = 57.12, MSE = 0.39, *p* < .001, 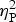 = .58, in which upright faces (*M* = 1.47, SE = 0.10) were recognised more accurately than inverted faces (*M* = 0.75, SE = 0.08) representing the standard face-inversion effect. These main effects were qualified by a significant interaction, *F*(1, 42) = 8.91, MSE = 0.41, *p* = .005, 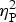 = .18. Bonferroni-corrected simple effects revealed that own-age upright faces were recognised more accurately than other-age upright faces, *t*(42) = 3.21, *p* = .006, Cohen’s *d* = 0.66; however, there was no difference between the recognition of inverted own- and other-age faces, *t*(42) = 0.06, *p* = .953, Cohen’s *d* = 0.01. We also ran correlations between participant age and recognition performance and found that the recognition of upright faces improved with age, *r*(41) = .40, *p* = .007, but the recognition of inverted faces did not, *r*(41) = .19, *p* = .218 (see also Hills, 2014).

**Table 1.** Mean (and standard error in parentheses), Hit rate, false alarm (FA) rate, recognition accuracy (*d*′), and response bias (*C*) of own- and other-age faces split by orientation.

			Face age	
			Own-age	Other-age
Hit rate	Facial orientation	Upright	0.81 (0.01)	0.66 (0.04)
		Inverted	0.61 (0.03)	0.54 (0.04)
FA rate	Facial orientation	Upright	0.22 (0.02)	0.30 (0.03)
		Inverted	0.35 (0.03)	0.28 (0.02)
*d′*	Facial orientation	Upright	1.76 (0.05)	1.19 (0.18)
		Inverted	0.75 (0.10)	0.75 (0.12)
*C*	Facial orientation	Upright	−0.05 (0.04)	0.07 (0.06)
		Inverted	0.06 (0.07)	0.26 (0.08)

**Figure 1.**
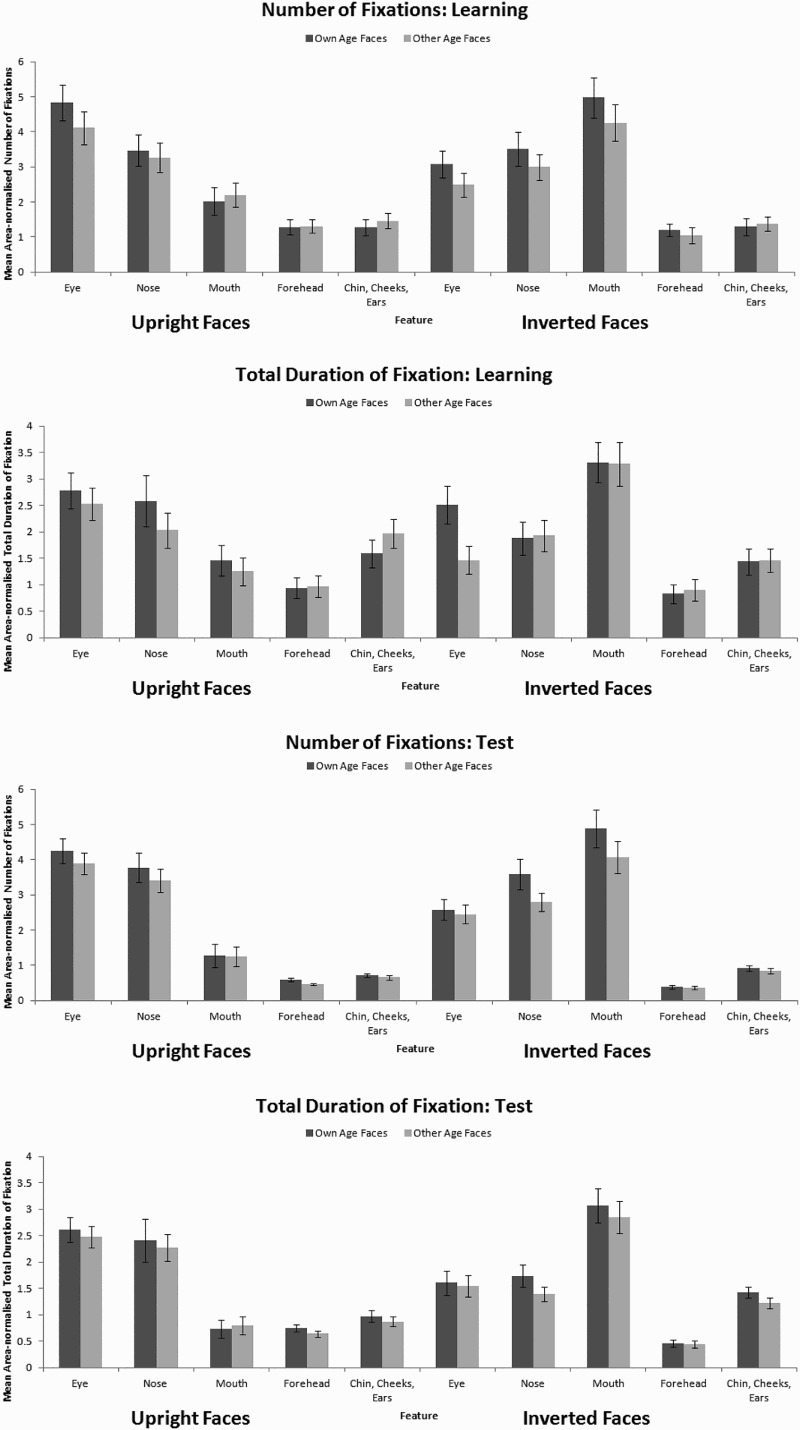
Mean (with standard error bars) fixation count and total fixation duration to each AOI for upright and inverted own- and other-age faces at learning (top panels) and at test (bottom panels).

The eye-tracking data were subject to parallel 2 × 2 × 5 within-subjects ANOVAs (see Figure 1) with the factors: face age (own- and other-age); orientation (upright and inverted) and facial feature (forehead and hair, eyes, nose, mouth, and chin and cheeks). We analysed the learning phase separately from the test phase data as the tasks require different processes. For all analyses involving the factor feature, Mauchley’s test of sphericity was significant, therefore the Huynh-Feldt correction was applied. Both the number of fixations and total duration of fixation data revealed that the internal features were scanned more than the external features at learning, *F*(3.34, 140.27) = 32.86, MSE = 7.71, *p* < .001, 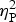 = .44 (number) and *F*(3.75, 157.65) = 15.48, MSE = 4.32, *p* < .001, 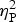 = .27 (duration) and at test, *F*(2.72, 114.76) = 88.39, MSE = 5.81, *p* < .001, 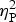 = .68 (number) and *F*(2.87, 120.45) = 31.99, MSE = 3.09, *p* < .001, 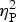 = .43 (duration). There was no difference in the scanning of the eyes, nose, and mouth (all *p*s > .146, Cohen’s *d*_s_ < 0.99); all three were scanned more than the chin, cheeks, and ears, and the forehead and hair (all *p*s < .001, Cohen’s *d*_s_ > 8.70). Inverted faces were scanned more often than upright faces at test, *F*(1, 42) = 4.87, MSE = 3.02, *p* = .033, 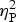 = .10 (number) but not for longer, *F*(1, 42) = 3.14, MSE = 1.12, *p* = .100, 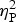 = .06 (duration). These effects interacted at learning, *F*(2.28, 95.65) = 7.27, MSE = 16.30, *p* = .001, 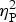 = .15 (number) and *F*(3.25, 136.62) = 10.12, MSE = 5.84, *p* < .001, 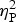 = .19 (duration) and at test, *F*(1.43, 60.17) = 18.82, MSE = 20.18, *p* < .001, 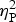 = .31 (number) and *F*(1.81, 76.09) = 20.45, MSE = 7.60, *p* < .001, 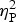 = .33 (duration). This interaction was such that there was no difference in the scanning of all features between upright and inverted faces except the mouth. The mouth was scanned significantly more in inverted faces than upright faces at learning, *t*(42) = 3.89, *p* < .001, Cohen’s *d* = 1.20 (number) and, *t*(42) = 4.35, *p* < .001, Cohen’s *d* = 1.34 (duration), and at test, *t*(42) = 5.36, *p* < .001, Cohen’s *d* = 1.65 (number) and *t*(42) = 6.43, *p* < .001, Cohen’s *d* = 1.98 (duration).

We also found that own-age faces were scanned more than other-age faces at learning, *F*(1, 42) = 4.08, MSE = 5.50, *p* = .050, 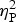 = .09 (number) and at test, *F*(1, 41) = 7.88, MSE = 2.22, *p* = .008, 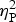 = .16 (number) and *F*(1, 38) = 6.35, MSE = 0.74, *p* = .016, 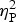 = .14 (duration). This effect did not interact with feature at learning, *F*(3.44, 144.52) = 0.59, MSE = 4.32, *p* = .648, 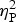 = .01 (number) and *F*(3.66, 153.59) = 1.37, MSE = 3.64, *p* = .248, 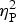 = .03 (duration) nor at test, *F*(2.81, 115.02) = 1.57, MSE = 1.83, *p* = .204, 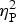 = .04 (number) and *F*(2.43, 92.21) = 0.70, MSE = 0.91, *p* = .525, 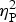 = .02 (duration), indicating that own- and other-age faces were scanned in a similar manner. To confirm this null result, we used Bayesian statistics comparing the values obtained here to those that would be predicted if the OAB was based on the same mechanisms as the face-inversion effect (i.e. that the orientation by facial feature interaction would produce an effect of a similar size as this face age by facial feature interaction). Bayes factors assess strength of evidence with a *B* of 1/3 or less indicating evidence for the null hypothesis rather than alternative hypothesis and a *B* of 3 or more indicating evidence for the alternative hypothesis rather than the null (see Dienes, 2014). Using the Bayes Factor calculator provided by Dienes (2015), we found that our data produced convincing evidence for the null hypothesis at learning, *B*_H(0,1)_ = 0.18 (number) and *B*_H(0,1)_ = 0.03 (duration) and at test, *B*_H(0,1)_ = 0.02 (number) and *B*_H(0,1)_ = 0.01 (duration). These Bayes factors confirm that the data for the face age by facial feature interaction do not conform to that predicted if the mechanisms of this effect were the same as the orientation by facial feature interaction. No other effects nor interactions were significant (largest *F* = 1.19, smallest *p* = .283, largest 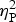 = .03).

**Figure 2.**
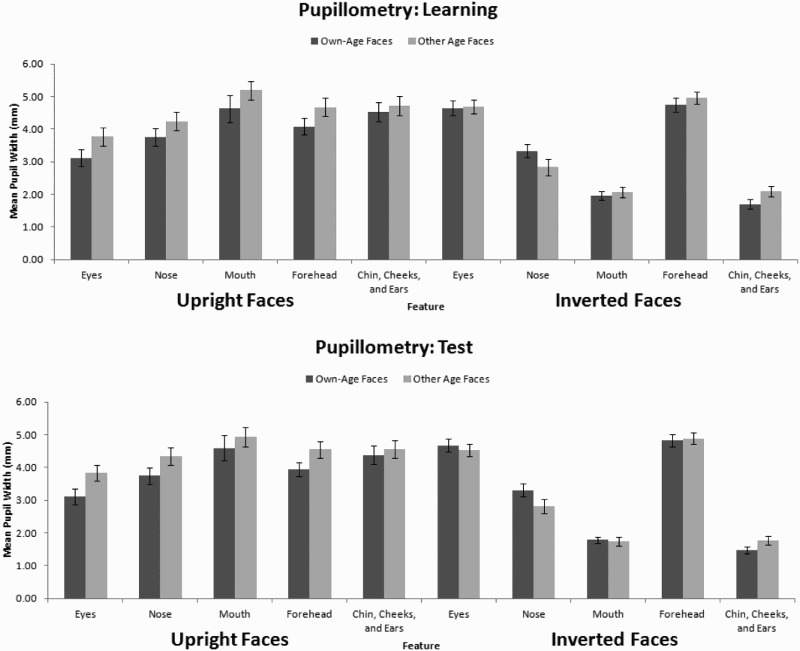
Mean (with standard error bars) pupil width when viewing upright and inverted own- and other-age faces, split by AOI at learning (top panel) and at test (bottom panel).

Pupillometry has been used as a measure of effort in cognitive processing during face recognition (e.g. Goldinger et al., 2009). The pupil width data were entered into a parallel 2 × 2 × 5 ANOVA (summarised in Figure 2), revealing that pupils were wider for upright (*M_l_* = 4.27, SE = 0.13 and *M_t_* = 4.20, SE = 0.15) than inverted faces (*M_l_* = 3.30, SE = 0.11 and *M_t_* = 3.18, SE = 0.11), at learning, *F*(1, 42) = 54.49, MSE = 3.71, *p* < .001, 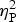 = .57 and at test, *F*(1, 42) = 58.61, MSE = 3.83, *p* < .001, 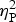 = .58. There was an effect of feature, at learning, *F*(3.38, 141.76) = 23.53, MSE = 2.59, *p* < .001, 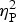 = .36 and at test, *F*(2.89, 121.54) = 33.41, MSE = 2.63, *p* < .001, 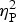 = .44. Pupils were wider for the eyes (*M_l_* = 4.06, SE = 0.16 and *M_t_* = 4.04, SE = 0.17) and forehead (*M_l_* = 4.61, SE = 0.14 and *M_t_* = 4.55, SE = 0.14) than the nose (*M_l_* = 3.54, SE = 0.14 and *M_t_* = 3.55, SE = 0.15), mouth (*M_l_* = 3.46, SE = 0.14 and *M_t_* = 3.27, SE = 0.14), and chin, cheeks, and ears (*M_l_* = 3.26, SE = 0.13 and *M_t_* = 3.05, SE = 0.13) (all *p*_s_ < .029, Cohen’s *d*_s_ > 1.13). These two effects interacted, at learning, *F*(3.90, 163.89) = 63.83, MSE = 2.37, *p* < .001, 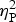 = .60 and at test, *F*(3.71, 155.81) = 92.13, MSE = 1.84, *p* < .001, 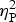 = .69. This interaction revealed that the pupil width was largely consistent when viewing upright faces. However, when viewing inverted faces, the pupils were much wider when viewing the eyes and forehead than the nose, mouth, chin, and cheeks.

Pupils were wider for other-age faces (*M_l_* = 3.93, SE = 0.13 and *M_t_* = 3.80, SE = 0.13) than own-age faces (*M_l_* = 3.65, SE = 0.11 and *M_t_* = 3.59, SE = 0.11), at learning, *F*(1, 42) = 4.28, MSE = 3.82, *p* = .045, 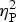 = .09 and at test, *F*(1, 42) = 4.10, MSE = 2.34, *p* = .049, 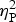 = .09. This effect interacted with orientation, at learning, *F*(1, 42) = 5.57, MSE = 1.95, *p* = .023, 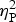 = .12 and at test, *F*(1, 42) = 8.41, MSE = 2.01, *p* = .006, 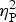 = .17. For upright faces, pupil dilation was greater for other-age (*M_l_* = 4.45, *M_t_* = 4.45) than own-age faces (*M_l_* = 4.02, *M_t_* = 3.96), at learning, *t*(42) = 2.23, *p* = .031, Cohen’s *d* = 0.69, and at test, *t*(42) = 2.70, *p* = .010, Cohen’s *d* = 0.83; for inverted faces, there does not appear to be a difference (compare *M_l_* = 3.15, *M_t_* = 3.15 with *M_l_* = 3.28, *M_t_* = 3.21), at learning, *t*(42) = 1.20, *p* = .239, Cohen’s *d* = 0.37, and at test, *t*(42) = 0.81, *p* = .424, Cohen’s *d* = 0.25.

## Discussion

Replicating the work from Hills (2012), we have shown that school-aged children show an OAB. Specifically, they show an OAB for upright faces but not inverted faces. This highlights that children employ expert face processing mechanisms for own- but not other-age faces. Replicating He et al. (2011) and Ebner et al. (2011), we found that own-age faces were scanned more than other-age faces, but other-age faces were more difficult to process. We will discuss each of these findings in turn, relating them to the theories of the OAB. We also found that upright and inverted faces were scanned differently, replicating Barton, Radcliffe, Cherkasova, Edelman, and Intriligator (2006, see also Hills, Sullivan, & Pake, 2012). Our pupillometry data indicate that upright faces were processed in a more effortful and deep manner.

Turning first to the OAB, we have found that children show an OAB for faces that are only a year or two different to their own age (Hills & Lewis, 2011). These results provide further evidence for constant changing and updating of the dimensions (Valentine, 1991) of face recognition system in children due to experience (Furl, Phillips, & O’Toole, 2002). However, our results indicate that children do not actively scan other-age faces differently to own-age faces: therefore they do not appear to be encoding different facial dimensions. Rather, they spend more time viewing them and making more fixations and thereby transitions between features (replicating Firestone et al., 2007). More time viewing faces has been taken as evidence for active interest in faces and therefore supporting the socio-cognitive motivational account of the OAB (He et al., 2011). Own-age faces are also processed in an expert manner (as revealed by the face-inversion effect for own-age faces). The deployment of expert, and presumably holistic processing, may be due to the motivation to process them appropriately. The expert processing afforded to own-age faces is coupled with more constricted pupils than employed for other-age faces, indicating fewer cognitive resources are being used to process them (Beatty, 1982; Granholm, Asarnow, Sarkin, & Dykes, 1996). This final finding is unexpected for two reasons: it is inconsistent with findings from the own-ethnicity bias found by Goldinger et al. (2009) and is apparently opposite to the findings regarding inversion.

There are a number of differences between the Goldinger study and ours that may explain this apparent conflict: while Sporer (2001) suggests that group biases in face recognition are based on the same mechanism, the OAB may be different to other biases because the type of faces that are other-age changes with one’s own age. Participants, therefore, lose their ability to recognise other-age faces rather than potentially never having it. Indeed, the pupil width data indicate that more effort was required to code younger faces (*M* = 4.78, SE = 0.25) than older faces (*M* = 4.28, SE = 0.14). A second reason for the difference in results is that they tested adults and we tested children. It is entirely possible for the mechanisms involved in the group biases to develop. Testing both group biases in children and adults would be necessary to establish whether the two biases are based on the same mechanism or not.

Returning to the central issue: own-age faces were scanned with apparently less cognitive effort than other-age faces. This may be due to more scanning of own-age faces than other-age faces leading to less effort required to encode each one. Alternatively, the expert processing mechanisms employed for own-age faces is highly efficient requiring less effort to form a holistic representation of own-age faces (theorised to be responsible for the processing of own-group faces, Michel, Rossion, Han, Chung, & Caldara, 2006). While this seems plausible, it does not fit with the obtained data that inverted faces required less effort to process, despite the face-inversion effect being a test of holistic processing (Freire, Lee, & Symons, 2000; Rossion, 2008). This pattern of results suggests that these two effects are based on different mechanisms employing different amounts of cognitive effort. Future work could experimentally manipulate, using different instructions, effort generally and effort to develop a holistic representation required to process own- and other-age faces to assess this claim. Alternatively, the recognition of own- and other-age faces could be equated for difficulty (by contrast-reversal, e.g.) and therefore the effort required to process them.

The results regarding the differences in the processing of upright and inverted faces indicate that inverting a face causes it to be encoded differently to upright faces. Firstly, the pupillometry data indicate that more interest was paid to upright faces relative to inverted faces. This result makes sense, given that upright faces are those that are more frequently encountered and are more socially relevant. Therefore, participants should be more interested in paying attention to upright faces. Indeed, it may indicate that expert coding afforded to upright faces is cognitively demanding. Expert perceptual processing is often based on chunking of specific spatial relations that are most frequently encountered (e.g. Newell & Simon, 1972). This may form the basis of holistic processing. Therefore, more information can be sampled quickly and efficiently. Inexpert processing, on the other hand, does not involve chunking, and individual features need to be attended to in order to integrate them into a meaningful whole (Rossion, 2008). This process is simpler but requires more fixations that are distributed across the whole face. This is precisely what we observed here.

When viewing upright faces, pupil width was largely consistent across all the features, indicating that all features were as easy to process or that a holistic representation was extracted from all features. Indeed, it has been established that early central fixations are all that are required to process faces (Hsiao & Cottrell, 2008). These fixations are sufficient to create a holistic representation of a face. Therefore, there is no need to process features with different levels of depth. When viewing inverted faces, pupil width was larger for the features that are normally the most fixated upon (Althoff & Cohen, 1999; Hills, Cooper, & Pake, 2013) and diagnostic (Hills, Ross, & Lewis, 2011): the eyes. This suggests that the participants are engaging in more effortful processing for the diagnostic features in inverted faces in order to enhance the representation of the face, potentially by increasing the effectiveness of featural processing.

Potentially, this final statement explains the differences in the pupillometry data between the OAB and the face-inversion effect. In order to process inverted faces, participants selectively deeply process the diagnostic features (the eyes). This creates an effect whereby the pupils are overall narrower due to averaging across the whole face. However, when processing own-age faces, efficient expert processing was employed. This highlights that it is easier to process own-age faces, potentially because we have more experience processing them. These results therefore indicate that the face-inversion effect is based on perceptual expertise, whereby eye-movements and pupillometry reveal patterns of viewing differences when looking at upright and inverted faces. However, the OAB appears to be based more on socio-cognitive mechanisms, since the differences that exist appear to be in interest paid to the face, rather than differential coding.

Finally, we should highlight that our results have serious implications for how face recognition is tested in children. Many studies investigating the development of face processing use adult faces (e.g. Carey & Diamond, 1977; Crookes & McKone, 2009). Using such inappropriate stimuli would necessarily reduce the reliability of such results: our data indicate that inexpert (and potentially not face-specific) mechanisms are used to process other-age faces. This means that to test the development of face recognition, age-matched stimuli must be used (see also Hills, 2012, 2014). Indeed, in our study, other-age stimuli were those approximately one year and greater age difference. This highlights the importance of close age-matching when testing face recognition in children (see also Firestone et al., 2007; Hills & Lewis, 2011).
